# Mesenchymal stem cells protect against ferroptosis via exosome-mediated stabilization of SLC7A11 in acute liver injury

**DOI:** 10.1038/s41419-022-04708-w

**Published:** 2022-03-26

**Authors:** Feiyan Lin, Wenyi Chen, Jiahang Zhou, Jiaqi Zhu, Qigu Yao, Bing Feng, Xudong Feng, Xiaowei Shi, Qiaoling Pan, Jiong Yu, Lanjuan Li, Hongcui Cao

**Affiliations:** 1 State Key Laboratory for the Diagnosis and Treatment of Infectious Diseases, Collaborative Innovation Center for Diagnosis and Treatment of Infectious Diseases, The First Affiliated Hospital, Zhejiang University School of Medicine, 79 Qingchun Rd., Hangzhou City, 310003 China; 2 National Clinical Research Center for Infectious Diseases, 79 Qingchun Rd., Hangzhou City, 310003 China; 3 Central Laboratory, The First Affiliated Hospital of Wenzhou Medical University, Wenzhou City, 325000 China; 4 Key Laboratory of Diagnosis and Treatment of Aging and Physic-chemical Injury Diseases of Zhejiang Province, 79 Qingchun Rd, Hangzhou City, 310003 China

**Keywords:** Hepatotoxicity, Experimental models of disease

## Abstract

Mesenchymal stem cells (MSCs) have attracted interest for their potential to alleviate liver injury. Here, the protective effect of MSCs on carbon tetrachloride (CCl_4_)-induced acute liver injury (ALI) was investigated. In this study, we illustrated a novel mechanism that ferroptosis, a newly recognized form of regulated cell death, contributed to CCl_4_-induced ALI. Subsequently, based on the in vitro and in vivo evidence that MSCs and MSC-derived exosomes (MSC-Exo) treatment achieved pathological remission and inhibited the production of lipid peroxidation, we proposed an MSC-based therapy for CCl_4_-induced ALI. More intriguingly, treatment with MSCs and MSC-Exo downregulated the mRNA level of prostaglandin-endoperoxide synthase 2 (*Ptgs2*) and lipoxygenases (*LOXs*) while it restored the protein level of SLC7A11 in primary hepatocytes and mouse liver, indicating that the inhibition of ferroptosis partly accounted for the protective effect of MSCs and MSC-Exo on ALI. We further revealed that MSC-Exo-induced expression of SLC7A11 protein was accompanied by increasing of CD44 and OTUB1. The aberrant expression of ubiquitinated SLC7A11 triggered by CCl_4_ could be rescued with OTUB1-mediated deubiquitination, thus strengthening SLC7A11 stability and thereby leading to the activation of system X_C_^−^ to prevent CCl_4_-induced hepatocyte ferroptosis. In conclusion, we showed that MSC-Exo had a protective role against ferroptosis by maintaining SLC7A11 function, thus proposing a novel therapeutic strategy for ferroptosis-induced ALI.

## Abstract (web summary)

Schematic diagram of the protective effect of MSC-derived exosomes on maintaining SLC7A11 function during ferroptosis involved in CCl_4_-induced ALI.

## Graphical abstract

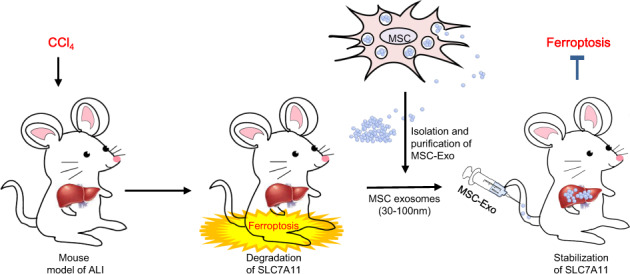

## Introduction

The liver has antibacterial/antiviral and drug detoxification functions and plays a central role in regulating homeostasis [1]. Acute liver injury (ALI) is one of the most prominent causes of liver diseases and is associated with high morbidity and mortality [2]. A variety of hepatotoxic factors, including viruses, lipid deposition, and drugs, can induce ALI, and 3.5% of deaths worldwide result from liver diseases [3, 4]. Therefore, it is crucial to elucidate the underlying mechanisms of ALI and to develop appropriate therapeutic strategies. Carbon tetrachloride (CCl_4_) is a typical hepatotoxic compound that is widely used to induce the ALI model for mechanism research [5]. Herein, we aimed to explore the potential mechanisms of ALI using a CCl_4_-induced mouse model and to explore possible effective treatment strategies.

Much attention has been focused on applying mesenchymal stem cells (MSCs) in biomedicine based on their potential in promoting liver regeneration and repairing liver injury. MSCs are a heterogeneous subset of stromal cells that can be easily isolated from adipose, bone marrow, and synovial tissues and can subsequently differentiate into numerous cell lineages according to specific biomedical applications. The immunological properties of MSCs, including their immune-regulatory, anti-inflammatory, and immunosuppressive abilities, suggest a potential role in immune tolerance [6]. Due to these properties, MSCs are considered ideal cell sources for tissue regeneration. Previously, we showed that MSCs transplantation attenuated CCl_4_-induced ALI by modulating the hepatic immune system [7]. Initial research suggested that MSCs exerted their therapeutic effects by engrafting to the site of injury. However, further studies showed that only a small percentage of injected MSCs reached their targets, whereas extracellular vesicles such as exosomes released by MSCs have vital and beneficial effects during treatment [8, 9]. In addition, exosomes released from MSCs contain special types of RNAs, lipids, and proteins, which are prerequisites for physiological homeostasis, cell proliferation, and tissue regeneration [10, 11]. Thus, we envision that MSC-derived exosomes (MSC-Exo) exhibit similar functions as MSCs and can be applied as MSC-based cell-free therapy.

In this study, CCl_4_ not only induced acute injury but simultaneously promoted ferroptosis in the liver. The hepatotoxic effects of CCl_4_ were related to its metabolic conversion in the liver to reactive intermediates. In addition, lipid peroxidation stimulation was observed in the liver shortly after administering CCl_4_ to mice in vivo. Of note, a high accumulation of products from lipid peroxidation initiated ferroptosis. In addition, various studies suggested that ferroptosis might be a new type of cell death associated with liver diseases such as viral hepatitis, drug-induced liver injury, alcoholic liver disease, non-alcoholic steatohepatitis, and hemochromatosis [12–15].

Ferroptosis is a newly identified form of regulated cell death driven by perturbation of the glutathione (GSH)-dependent lipid-hydroperoxide-scavenging network [16, 17]. Lipid peroxidation is regulated by system X_C_^−^, which transports glutamate out of the cell in exchange for the transport of cysteine, an important precursor for GSH synthesis, into the cell [18]. System X_C_^−^ consists of a light-chain subunit (SLC7A11/xCT) and a heavy-chain subunit (SLC3A2). SLC7A11 exhibits transporter activity highly specific for glutamate and cysteine and thus plays an important role in providing cysteine for the biosynthesis of GSH. GSH is vital for glutathione peroxidase 4 (GPX4), which protects cells against ferroptosis by converting toxic lipid hydroperoxides into nontoxic lipid alcohol [19]. Emerging strategies targeting ferroptosis have been developed to treat organ and tissue injury [20–22]. Furthermore, it is reported that MSC-Exo alleviates oxidative stress-induced dysfunction in mouse livers in vivo [23].

Hence, in this study, we investigated the role of ferroptosis in MSC-based treatment for ALI in vitro and in vivo. Our results showed that SLC7A11 was significantly downregulated in the ALI mouse model, and its down-regulation promoted CCl_4_-induced hepatocyte ferroptosis. MSC transplantation and MSC-Exo treatment largely abolished ferroptosis and protected the liver from injury. Moreover, MSC-Exo-mediated recovery of SLC7A11 protein was accompanied by the increase of CD44 and OTUB1 in ALI mouse livers. The stability of SLC7A11 was closely related to OTUB1-mediated deubiquitination. Finally, our studies indicated that MSC-Exo suppressed hepatocyte’s ferroptosis to mediate liver repair in ALI mice by maintaining SLC7A11 function.

## Results

### Ferroptosis drove CCl_4_-induced mouse models of ALI

**Fig. 1:**
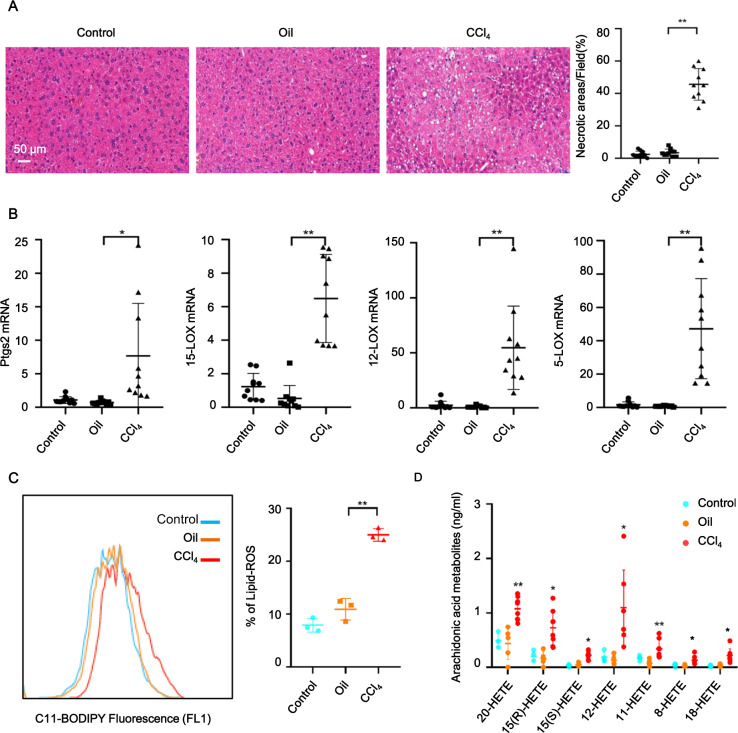
Ferroptosis occurred in livers of ALI mouse models. **A** Hematoxylin and eosin (HE) staining showed that administration of CCl_4_ induced ALI in mice. **B** *Ptgs2, 15-LOX, 12-LOX*, and 5-*LOX* mRNA levels were measured in livers of 8-week-old male C57BL/6 mice treated for 6 h with or without CCl_4_. mRNA levels were normalized to that of GAPDH and were expressed relative to the mean value in the PBS-treated mice. **C** Lipid peroxidation was measured using C11-BODIPY^581/591^ staining. **D** Ultra-performance liquid chromatography-tandem mass spectrometry (UPLC-MS/MS) analysis of 20-hydroxyeicosatetraenoic acid (HETE), 15(R)-HETE, 15(S)-HETE, 12-HETE, 11-HETE, 8-HETE, and 18-HETE were performed in liver tissues. Significance was calculated using one-way ANOVA with Tukey’s post hoc test. *p < 0.05 or **p < 0.001 indicated a significant difference between groups.

As shown in Fig. 1A, compared with control (PBS) and oil-treated groups (left and middle), typical histopathological changes of ALI, such as diffuse hepatic necrosis, were observed microscopically in the liver tissues from CCl_4_-treated groups. To investigate the contribution of ferroptotic cell death in CCl_4_-induced ALI, we performed real-time PCR analysis of putative molecular markers of ferroptosis and found that CCl_4_ treatment robustly induced increased mRNA levels of liver prostaglandin-endoperoxide synthase 2 (*Ptgs2), 15-LOX, 12-LOX*, and *5-LOX* (Fig. 1B). Next, we quantified lipid peroxidation in living liver cells by flow cytometry using the C11-BODIPY^581/591^ fluorescent probe, a canonical index of ferroptosis. Compared to PBS and oil treatments, the lipid-ROS levels in mouse livers were significantly elevated after CCl_4_ treatment (Fig. 1C). The release of an oxidized lipid mediator is a reported characteristic of ferroptosis [24]. Membranes with arachidonic acid enrichment may facilitate ferroptosis by releasing arachidonic acid metabolites (hydroxyeicosatetraenoic acid) during cell death. Interestingly, the levels of 20-hydroxyeicosatetraenoic acid (HETE), 15(R)-HETE, 15(S)-HETE, 12-HETE, 11-HETE, 8-HETE, and 18-HETE were increased in the livers from CCl_4_-treated mice (Fig. 1D). These findings suggested that ferroptosis is a crucial driver of CCl_4_-induced liver injury in mice.

### MSC transplantation alleviated CCl_4_-induced ferroptosis in ALI

**Fig. 2:**
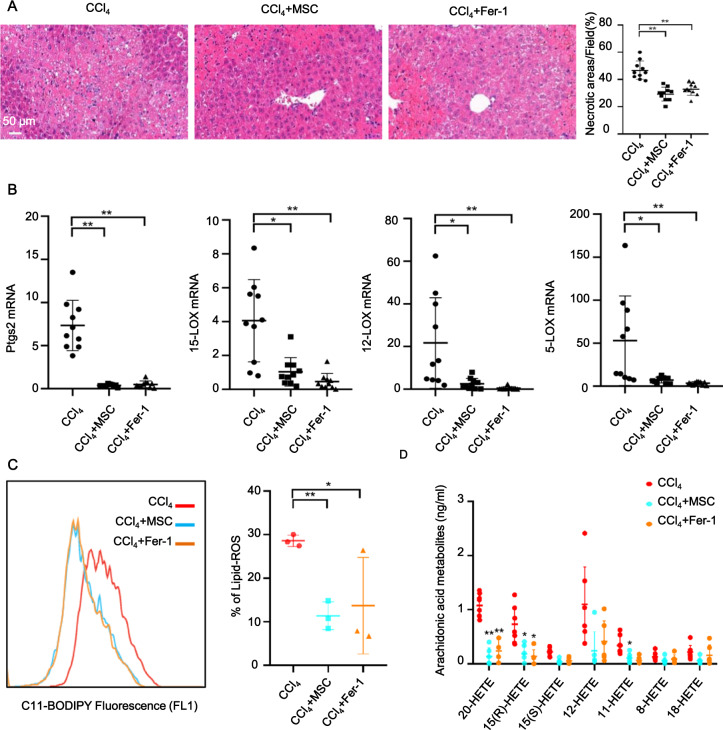
MSC treatment prevented CCl_4_-induced ferroptosis in ALI. **A** HE staining showed that MSC and ferrostatin-1 (Fer-1) treatment alleviated CCl_4_-induced ALI. **B** *Ptgs2, 15-LOX, 12-LOX*, and *5-LOX* mRNA levels were measured in mouse livers of CCl_4_, MSC, and Fer-1 groups. mRNA levels were normalized to that of GAPDH. **C** Lipid peroxidation was measured by C11-BODIPY^581/591^ staining. Lipid peroxidation of hepatocytes in the liver was significantly increased in the CCl_4_ group but significantly reduced in MSC and Fer-1 groups. **D** UPLC-MS/MS detection showed increased levels of 20-hydroxyeicosatetraenoic acid (HETE), 15(R)-HETE, 15(S)-HETE, 12-HETE, 11-HETE, 8-HETE, and 18-HETE in mouse livers of the CCl_4_ group, and these effects were abrogated by MSC and Fer-1 treatment. Significance was calculated by one-way ANOVA with Tukey’s post hoc test. *p < 0.05 or **p < 0.001 indicated a significant difference between groups.

We have previously reported that MSC treatment dramatically alleviated CCl_4_-induced ALI due to its immunomodulatory function [7]. Given that MSCs also play important roles in reducing oxidative stress [25], and oxidative stress-induced excessive accumulation of lipid hydroperoxides ultimately results in ferroptosis, we hypothesized that MSCs might exert a protective effect against ferroptosis by facilitating lipid-ROS scavenging in CCl_4_-induced ALI. Histopathological staining indicated that hepatic necrosis was drastically mitigated following administration of MSCs and ferrostatin-1(Fer-1, a ferroptosis inhibitor), demonstrating the therapeutic effect of MSCs against CCl_4_-induced ferroptosis in ALI (Fig. 2A). We observed significant decreases in mRNA levels of classic biomarkers of ferroptosis, including *Ptgs2, 15-LOX, 12-LOX*, and *5-LOX* in MSC and Fer-1 groups (Fig. 2B). MSC treatment also potently reduced the accumulation of CCl_4_-induced lipid hydroperoxides in the liver (Fig. 2C). Notably, MSCs exhibited comparable therapeutic efficacy to ferrostatin-1, as evidenced by the similar trends in downregulating mRNA levels of liver *Ptgs2* and *LOXs*, and lipid peroxidation. It is worth noting that *LOXs* (particularly *15-LOX*) have been reported as essential regulators of ferroptotic cell death as they contribute to the cellular pool of lipid hydroperoxides [26, 27]. Moreover, the increased levels of 20-hydroxyeicosatetraenoic acid (HETE), 15(R)-HETE, 15(S)-HETE, 12-HETE, 11-HETE, 8-HETE, and 18-HETE induced by CCl_4_ were abrogated by MSC and Fer-1 treatment (Fig. 2D). These results suggested that the potential ability of MSCs to down-regulate peroxidation resulted in lipid-ROS reduction and inhibited the progression of ferroptosis.

### SLC7A11 protein level was reduced in CCl_4_-induced ALI but increased following MSC treatment in vivo and in vitro

**Fig. 3:**
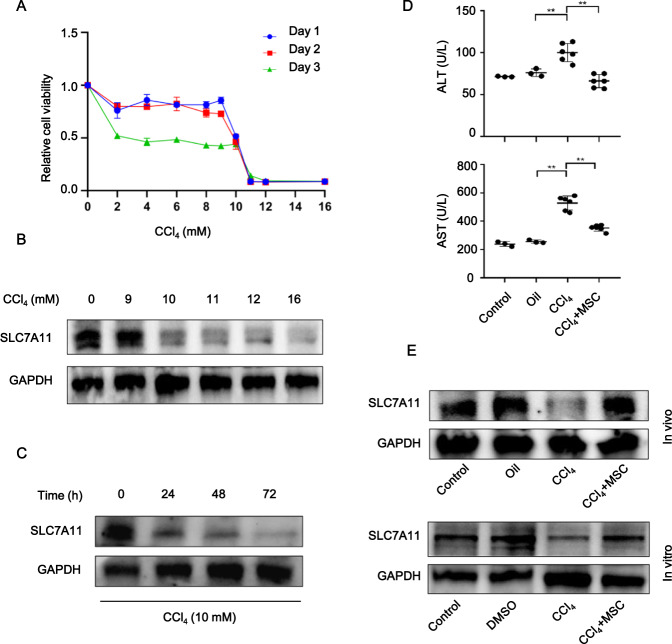
SLC7A11 protein level was downregulated in CCl_4_-induced ALI and upregulated following MSC treatment in vivo and in vitro. **A** Cell viability was measured on days 1, 2, and 3, after hepatocytes were treated with 0, 2, 4, 6, 8, 9, 10, 11, 12, and 16 mM CCl_4_. **B** WB analysis was performed to measure the SLC7A11 protein level in mouse primary hepatocytes treated with 0, 9, 10, 11, 12, and 16 mM CCl_4_ for 48 h. **C** or with 10 mM CCl_4_ for 0, 24, 48, and 72 h. **D** Coculture of primary hepatocytes with MSCs significantly reduced AST and ALT induced by CCl_4_. **E** MSC treatment significantly reduced the downregulated SLC7A11 protein level induced by CCl_4_ in vivo and in vitro. Significance was calculated by one-way ANOVA with Tukey’s post hoc test. *p < 0.05 or **p < 0.001 indicated a significant difference between groups.

Next, we established an in vitro acute hepatocyte injury model to further investigate the therapeutic mechanism of MSCs in ALI treatment. On days 1, 2, and 3, a dramatic decrease in cell viability was detected using the Cell Counting Kit-8 when CCl_4_ concentration was increased to 10 mM (Fig. 3A). To excavate the role of SLC7A11 protein in the CCl_4_-induced acute hepatocyte injury model, we performed WB analysis to assess the dose- and time-dependent effects of CCl_4_ treatment on SLC7A11 expression. SLC7A11 protein level was significantly downregulated at the CCl_4_ concentration of 10 mM (Fig. 3B and Fig. S1A). Furthermore, the decreased SLC7A11 protein levels were detected at 24, 48, and 72 h after CCl_4_ treatment (10 mM) (Fig. 3C and Fig. S1B).

As previous studies concluded that only a small percentage of injected MSCs reached their targets, we speculated that some active substances derived from MSCs might participate more profoundly in alleviating ALI. Recent studies have shown that MSCs produce exosomes, which can ameliorate tissue injury via the delivery of their DNA, miRNA, and protein contents [28–30]. Given this, we hypothesized that these extracellular vesicles were actively facilitated the protective effects of MSCs against ferroptosis. Subsequent to the above primary hepatocyte model, a coculture system, where MSCs were physically separated from damaged hepatocytes by transwell chambers, was exploited to investigate whether the therapeutic efficacy of MSCs can be achieved. As shown in Fig. 3D, AST and ALT levels were significantly increased in the CCl_4_ group but then dramatically reduced after MSCs coculture. Next, to ascertain whether coculture with MSCs can also rescue the CCl_4_-induced decrease in SLC7A11 protein level in primary hepatocytes, WB analysis was performed. Interestingly, similar to the in vivo findings, SLC7A11 protein level was reduced in the CCl_4_ group compared with the PBS group but was restored by MSCs coculture (Fig. 3E and Fig. S1C, D). These results suggested that something like the exosome derived from MSCs mediated the regulation of SLC7A11 protein levels to facilitate the protective effect of MSCs against ferroptosis.

### MSC-Exo inhibited ferroptosis in CCl_4_-induced ALI in vitro

**Fig. 4:**
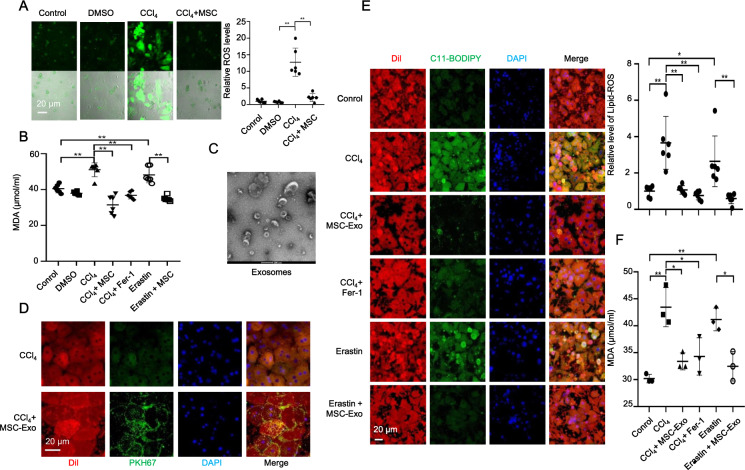
MSC-Exo mediated the effect of MSCs against CCl_4_-induced ferroptosis in vitro. **A** Relative ROS levels were measured by H_2_DCFDA staining in primary hepatocytes treated with control (PBS), dimethyl sulfoxide (DMSO), or 10 mM CCl_4_ for 48 h with or without MSCs coculture. **B** The increased MDA level induced by CCl_4_ was downregulated by MSCs coculture and Fer-1 treatment in primary hepatocytes. The increased MDA level induced by erastin was also downregulated by MSCs coculture in primary hepatocytes. **C** Refrigerated transmission electron microscopy was used to assess exosomes isolated from MSCs. **D** Exosomes collected from MSC-conditioned medium were labeled with PKH67 and incubated with CCl_4_-induced acute injured hepatocytes for 24 h. The cell membrane was stained with Dil. The nuclei were stained with DAPI. PKH67 lipid dye was detected in damaged hepatocytes treated with PKH67-labeled exosomes in the CCl_4_ group. **E** Isolated hepatocytes were incubated with C11-BODIPY^581/791^ and then analyzed by using laser confocal focus. Cell membrane was stained with Dil. The nuclei were stained with DAPI. C11-BODIPY^581/591^ staining showed a significant increase in lipid peroxidation in hepatocytes following CCl_4_ and erastin treatment, while MSC-Exo and Fer-1 downregulated the increased lipid peroxidation induced by CCl_4_ and erastin. **F** The increased MDA level induced by CCl_4_ and erastin were downregulated by MSC-Exo and Fer-1 treatments in primary hepatocytes. Significance was calculated by one-way ANOVA with Tukey’s post hoc test. *p < 0.05 or **p < 0.001 indicated a significant difference between groups.

To further examine the role of MSCs in ALI, we quantified the levels of ROS and MDA. As shown in Fig. 4A, MSCs coculture reduced ROS accumulation induced by CCl_4_ in hepatocytes. In addition, CCl_4_ induced a dramatic increase of MDA, similar to erastin (a ferroptosis inducer), while coculture with MSCs, similar to Fer-1 treatment, downregulated MDA level both in CCl_4_ and Erastin group in hepatocytes (Fig. 4B).

Next, we extracted and identified exosomes from an MSC-conditioned medium. We used refrigerated transmission electron microscopy (TEM) (Fig. 4C) and WB analysis to characterize vesicles recovered from the MSC-conditioned medium by differential ultracentrifugation and identified 30–100 nm vesicles that were morphologically similar to exosomes, which expressed the exosomal markers of CD63 and CD81 (Figs. S2A, S3A). To determine whether hepatocytes can internalize MSC-Exo, exosomes were manually labeled with green lipophilic fluorescent dye PKH67 (PKH67GL; Sigma-Aldrich), and were subsequently cocultured with CCl_4_-induced injury hepatocytes. The hepatocytes exhibited high uptake efficiency, as indicated by the strong green fluorescent signal (Fig. 4D). Next, we measured the lipid peroxidation in CCl_4_-treated primary hepatocytes with or without MSC-Exo treatment. Similar to erastin, CCl_4_ induced significant upregulation of lipid-ROS and MDA levels, whereas MSC-Exo or Fer-1 treatment comparably decreased the level of lipid-ROS (Fig. 4E) and MDA (Fig. 4F). These data suggested that MSC-Exo inhibited ferroptosis in CCl_4_-induced ALI in vitro.

### MSC-Exo inhibited ferroptosis in CCl_4_-induced ALI in vivo

**Fig. 5:**
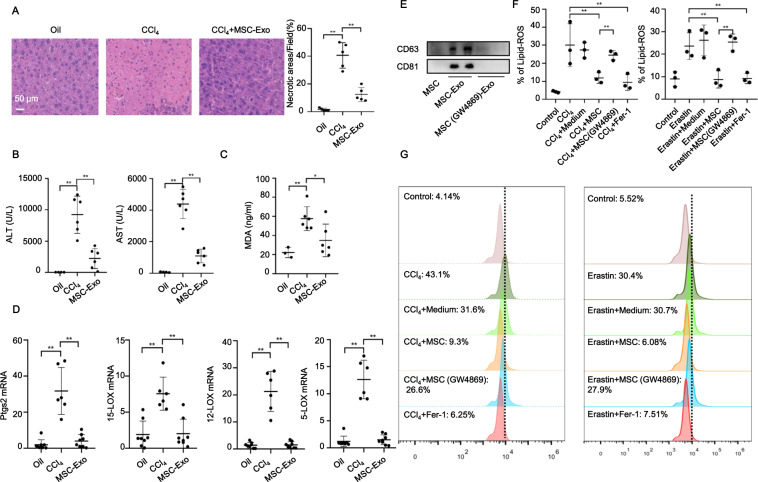
MSC-Exo mediated the effect of MSCs against CCl_4_-induced ferroptosis in vivo. **A** HE staining showed that MSC-Exo treatment alleviated CCl_4_-induced ALI. **B** MSC-Exo dramatically reversed the increased serum levels of ALT and AST induced by CCl_4_. **C** UPLC-MS/MS detection indicated significant upregulation of the serum MDA level induced by CCl_4_, which was downregulated following MSC-Exo treatment. **D** MSC-Exo treatment significantly reversed the increased mRNA levels of *Ptgs2*, *15-LOX*, *12-LOX*, and *5-LOX* induced by CCl_4_ in the liver. **E** WB analysis of CD63 and CD81 in exosomes extracted from MSC-conditioned medium and GW4869 pretreated MSC-conditioned medium. **F** Lipid peroxidation was measured using C11-BODIPY^581/591^ staining in L-02 cells treated with CCl_4_, CCl_4_ + Medium, CCl_4_ + MSC, CCl_4_ + MSC (pretreated with GW4869), and CCl_4_ + Fer-1 (left panel). After replacing CCl_4_ with erastin, the above experiment was repeated three times (right panel). **G** Representative histograms of C11-BODIPY^581/591^ staining. Significance was calculated by one-way ANOVA with Tukey’s post hoc test. *p < 0.05 or **p < 0.001 indicated a significant difference between groups.

Inspired by the in vitro potency of MSC-Exo, we next investigated the effects of MSC-Exo in CCl_4_-induced ferroptosis in vivo. Interestingly, consistent with the protective effects of MSCs in vivo, MSC-Exo also ameliorated hepatic necrosis and downregulated the aberrant ALT/AST in CCl_4_-induced ALI (Fig. 5A, B). In addition, as evidenced by MDA quantification and real-time PCR analysis of ferroptosis-related markers, MSC-Exo treatment significantly downregulated MDA levels and mRNA levels of liver *Ptgs2, 15-LOX, 12-LOX*, and *5-LOX* (Fig. 5C, D). These findings suggested that MSC-Exo also inhibited ferroptosis in CCl_4_-induced ALI in vivo.

To confirm the anti-ferroptotic effect of MSC-Exo, we used GW4869 to inhibit the release of exosomes in MSCs. WB analysis revealed that the secretion of exosomes was significantly inhibited by GW4869 in MSCs (Fig. 5E and Fig. S4). As shown in Fig. 5F, G, MSC pretreated with GW4869 (10 µM) showed limited potency in downregulating lipid-ROS levels. What’s more, in vivo study also demonstrated that MSC pretreated with GW4869 showed limited potency in downregulating lipid-ROS levels as evidenced by 4-hydroxynonenal (4-HNE; another marker of lipid peroxidation) detection (Fig. S5).

### MSC-Exo protected against ferroptosis via stabilization of SLC7A11 in ALI

Next, we investigated how MSC-Exo realized its protective potency against CCl_4_-induced ferroptosis. Here, more focus was paid to the dynamic regulation of SLC7A11 protein. Accumulating evidence indicated that SLC7A11 was modulated by CD44 [18, 31], a cell surface marker of MSCs [7] and a critical molecule for MSCs recruitment to CCl_4_-induced ALI livers by the firm adhesion between MSCs and injured liver tissues [32]. Desirably, we detected CD44 protein expression in MSC-Exo (Figs. S2A, S3A). In the above study, compared to healthy mice in the PBS group, a reduced SLC7A11 protein level was detected among CCl_4_-induced ALI mice but can be restored after MSC treatment. Therefore, we hypothesized that the SLC7A11 level was functionally related to the anti-ferroptotic effects of MSC-Exo treatment.

**Fig. 6:**
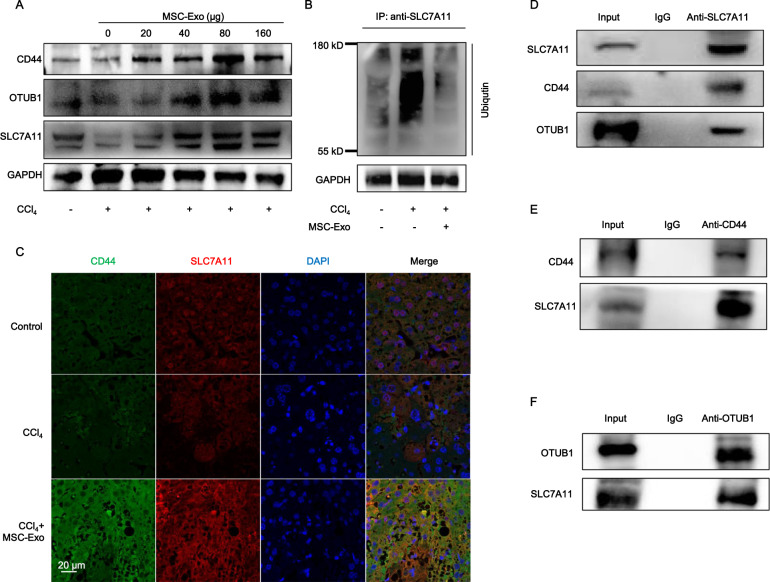
MSC-Exo protected against ferroptosis via the stabilization of SLC7A11 in ALI. **A** CD44, OTUB1, and SLC7A11 protein levels in CCl_4_-induced acute injured hepatocytes treated with 0, 20, 40, 80, and 160 µg MSC-Exo were detected by WB analysis. **B** CCl_4_-induced ALI increased ubiquitination of SLC7A11 in hepatocytes, and this effect was downregulated following MSC-Exo treatment. **C** Immunofluorescence assay was used to detect the protein levels of CD44 and SLC7A11 in liver tissues. **D** WB analysis of CD44 and OTUB1 after co-immunoprecipitation of anti-SLC7A11 from liver tissue in CCl_4_ group treated with MSC-Exo. One percent of the sample was loaded as input. **E** WB analysis of SLC7A11 after co-immunoprecipitation of anti-CD44 from liver tissue in CCl_4_ group treated with MSC-Exo. One percent of the sample was loaded as input. **F** WB analysis of SLC7A11 after co-immunoprecipitation of anti-OTUB1 from liver tissue in CCl_4_ group treated with MSC-Exo. One percent of the sample was loaded as input. Significance was calculated by one-way ANOVA with Tukey’s post hoc test. *p < 0.05 or **p < 0.001 indicated a significant difference between groups.

As shown in Fig. 6A (Fig. S6A), SLC7A11 protein levels in acute injured hepatocytes treated with 0, 20, 40, 80, and 160 µg MSC-Exo were measured by WB analysis. Interestingly, MSC-Exo-induced recovery of SLC7A11 protein was accompanied by increasing of CD44 and OTUB1. What’s more, MSC-Exo treatment also induced recovery of SLC7A11 and CD44 in vivo (Figs. S2B, S3B). OTUB1 is an ovarian tumor deubiquitinase family member with reported functions to regulate SLC7A11 stability via direct protein interaction [33]. Accordingly, we found that CCl_4_ administration increased the ubiquitination of SLC7A11, while MSC-Exo treatment downregulated the ubiquitination of SLC7A11 (Fig. 6B and Fig. S6B). The above data suggested that CCl_4_-induced ubiquitination abolished the stabilization of SLC7A11, leading to a downregulated level of SLC7A11 in ALI. However, MSC-Exo treatment restored the stabilization of SLC7A11 by inhibiting its ubiquitination, resulting in upregulation of SLC7A11 in ALI.

To determine the mechanism by which MSC-Exo exerted protective effects against ferroptosis, we performed immunofluorescence and co-immunoprecipitation analyses. Immunofluorescence analysis suggested that CD44 and SLC7A11 were co-expressed and increased in ALI after MSC-Exo treatment compared with CCl_4_ and PBS groups (Fig. 6C). Next, to measure the interaction under physiological conditions, we performed co-immunoprecipitation analysis targeting endogenous proteins expressed in liver tissues of ALI after MSC-Exo treatment. As shown in Fig. 6D (Fig. S6C), the endogenous CD44 and OTUB1 proteins were co-precipitated by a SLC7A11-specific antibody, while endogenous SLC7A11 was co-precipitated by a CD44-specific and OTUB1-specific antibody, respectively (Fig. 6E, F and Fig. S6D, E). Thus, we further speculated that MSC-Exo treatment promotes the increase of CD44 and OTUB1 proteins and stabilizes SLC7A11 by directly interacting with and reducing its ubiquitination in CCl_4_-induced ALI.

### MSC-Exo in the circulation localized more readily in the injured liver

**Fig. 7:**
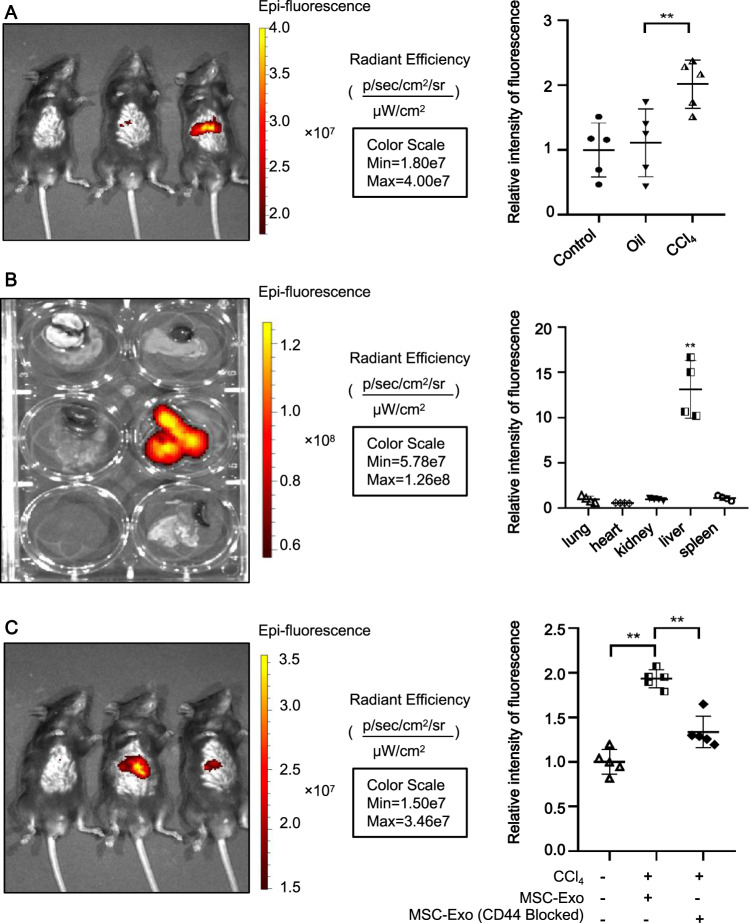
MSC-Exo in the circulation was more readily localized in the damaged liver. **A** Whole-body fluorescence imaging of C57BL/6 male mice treated with 8 mg/kg labeled MSC-Exo. Images were taken 6 h after tail vein injection. **B** Quantitative fluorescent imaging of lung, heart, kidney, liver, and spleen from recipient mice receiving labeled MSC-Exo. **C** Neutralizing antibodies against CD44 reduced hepatic engraftment of MSC-Exo in ALI murine livers. Images were taken at 6 h. Data were the mean ± SD fluorescence; *p < 0.05, **p < 0.001; *n* = 5. Individual images were presented.

Next, we assessed whether MSC-Exo was preferentially accumulated within injured livers. For this purpose, MSC-Exo was first isolated from an MSC-conditioned medium and then labeled with lipophilic carbocyanine DiR. After intravenous injection via tail vein to ALI mice, the bio-distribution of DiR-labeled MSC-Exo was visualized by an IVIS imaging system. From in vivo fluorescent imaging, the accumulation of DiR-labeled MSC-Exo was detected within the injured and healthy livers 6 h postinjection. However, stronger fluorescence was observed in livers from ALI mice compared to those from PBS or oil groups (Fig. 7A), indicating that circulating MSC-Exo was more likely to aggregate in damaged tissue sites. We also evaluated the fluorescence intensity within major organs including lungs, hearts, kidneys, livers, and spleens of ALI mice treated with DiR-labeled MSC-Exo. As shown in Fig. 7B, significantly higher fluorescent signals were observed in the liver compared with other organs, suggesting that MSC-Exo in circulation localized more readily in the livers in ALI mice. However, neutralizing antibodies against CD44 reduced MSC-Exo targeting to the diseased liver (*P* < 0.05) (Fig. 7C). These results indicated that the affinity of MSC-Exo to injured liver tissues was assisted by the binding function of CD44.

## Discussion

Ferroptosis is a newly recognized non-apoptotic form of regulated cell death characterized by an overwhelming accumulation of lipid hydroperoxides that contributes to a wide range of pathologies, including cancer, neurodegeneration, and tissue injury [16, 34, 35]. Accumulating evidence suggested that patients with liver diseases were likely to suffer from ferroptosis-regulated cell death [34, 36]. Recently, it was reported that ferroptosis also participated in liver fibrosis [37].

In this study, aside from the expansion of liver necrotic areas, lipid hydroperoxides were also dramatically upregulated within ALI livers, suggesting excessive accumulation of lipid-ROS. Furthermore, we observed increased expression of ferroptosis-relevant genes after CCl_4_ treatment such as *Ptgs2* and *LOXs. Ptgs2*, also known as cyclooxygenase-2, was induced in cells undergoing ferroptosis [19]. Thus, these results suggested that ferroptosis-mediated cell death occurred in CCl_4_-induced ALI. In addition, as ferrostatin-1 and MSC treatment exhibited similar therapeutic efficacy in downregulating lipid-ROS accumulation and ferroptosis-relevant gene expression in the ALI mouse model, our observations shed an intriguing light on the protective role of MSCs against ferroptosis. Further investigations should be conducted to fully elucidate this anti-ferroptotic mechanism of MSCs.

Recently, increasing evidence has shown that most of the MSC-mediated beneficial effects can be attributed to the functions of released exosomes. For instance, exosomes from human umbilical cord blood-derived MSCs can protect livers against ALI via intercellular communication [23]. Therefore, we further evaluated the hepatoprotective role of MSC-Exo. Inspiringly, our study revealed that MSC-Exo treatment resulted in equal alleviation as MSCs both in vivo and in vitro. To fully explore the underlying mechanism, we tested the effect of MSC-Exo on liver antioxidant systems and found that MSC-Exo significantly attenuated CCl_4_-provoked lipid peroxidation, as manifested by the levels of MDA and lipid ROS in liver tissues and hepatocytes. In addition, MSC-Exo also abolished other ferroptosis markers raised by CCl_4_ treatment, including decreased expressions of SLC7A11 and increased expressions of *Ptgs2* and *LOXs*. Thus, we concluded that MSC-Exo protected against CCl_4_-induced liver injury through inhibiting hepatocyte ferroptosis.

System X_C_^−^-mediated antioxidant defense can effectively protect cells and tissues against ferroptosis [38]. SLC7A11, a multi-pass transmembrane protein, mediates the cystine/glutamate antiporter activity in the system X_C_^−^. Once transported into the cell, cystine is rapidly converted to cysteine, which is the rate-limiting precursor of GSH. Inhibition of SLC7A11 has been reported to increase ferroptosis sensitivity [39]. Emerging evidence suggested that upregulation of SLC7A11 promoted cell growth by suppressing ferroptosis. Here, we showed that treatment with CCl_4_ reduced the protein level of SLC7A11 in mouse liver and primary hepatocytes. However, MSC-Exo efficiently restituted the aberrant level of ALT/AST and restored the SLC7A11 protein level in ALI mice. Thus, we concluded that MSC-Exo protected against CCl_4_-induced ALI through inhibiting hepatocyte ferroptosis via restoring the SLC7A11 protein level. Additionally, the exosome-induced recovery of SLC7A11 protein was accompanied by upregulations of CD44 and OTUB1. CD44 is a major adhesion molecule in the extracellular matrix and is involved in a variety of physiological processes, including stem cell homing, wound healing, and cell migration [40, 41]. Several studies have found that CD44 stabilizes SLC7A11 expression at the plasma membrane, with the System xc^−^ then mediating cystine uptake and promoting GSH synthesis [18]. And OTUB1, a deubiquitinase, has the function to regulate SLC7A11 stability via direct protein interaction. These data indicated that MSC-Exo might alleviate ferroptosis via the CD44 and OTUB1-mediated stabilization of SLC7A11. More interestingly, MSC-Exo in the circulation was preferentially localized in damaged livers. Since the same amount of MSC-Exo was injected, the observed difference in localization may be driven by factors intrinsic to MSC-Exo. The target specificity of exosomes is thought to be mediated by adhesion proteins on the surface of the vesicles [42]. Interestingly, we observed a high expression of CD44 both in MSCs and MSC-Exo. What’s more, it has been demonstrated that MSCs can adhere to liver tissue in diseased conditions. And these adhesive events are largely regulated by CD44 [32]. In this study, more fluorescence was detected in the damaged liver, suggesting that MSC-Exo had the same aggregation function as MSCs. In addition, liver localization of MSC-Exo was blocked by preincubation with CD44 blocking antibody. Thus, we suggest that CD44 recruit MSC-Exo to the injured liver and enhance liver recovery. But the underlying mechanism by which MSC-Exo restored the expression of CD44 in damaged hepatocytes remains further study. We prefer to speculate that hepatic uptake of CD44 expressing MSC-Exo promoted the accumulation of CD44 in hepatocytes. However, it is not excluded that other components in exosomes, such as miRNA, lncRNA, or other protein components, play a role in regulating CD44 protein levels. Taken together, this study has vital implications in illustrating how MSC-Exo-mediated anti-ferroptotic effects are achieved in ALI. However, the specific mechanism remains to be further studied.

## Materials and methods

### Preparation of MSCs and MSC-Exo

MSCs were obtained from compact bones of 1-week-old C57BL/6 male mice [7, 43]. MSCs from passages 3–5 were used in the experiments. Detailed information was shown in the supplementary materials.

MSCs were cultured in an exosome-free medium (with or without 10 µM GW4869, HY-19363; MedChemExpress, an exosome inhibitor), and exosomes were isolated and purified by ultracentrifugation. Briefly, MSC-conditioned medium was collected after 48 h of culture and centrifuged at 2000×*g* for 20 min to remove debris and cells. The supernatant was collected and transferred to a new sterile tube and centrifuged at 10,000×*g* for 15 min. The supernatant was filtered using a 0.22-µm-pore sterile filter, followed by ultracentrifugation at 110,000 × *g* for 70 min at 4 °C to obtain exosome pellets, which were resuspended in PBS and stored at −80 °C. We used the BCA protein assay kit (UD283191; Thermo Fisher Scientific, Waltham, MA, USA) to determine the protein content of the concentrated exosomes. Exosomes were identified by Western blot (WB) analysis of the marker proteins CD63 and CD81. The morphology of exosomes was observed using a 120 kV refrigerated transmission electron microscope (Tecnai Spirit Bio-TWIN, FEI Company, Hillsboro, OR, USA). The MSC-Exo concentration used in vivo was 8 mg/kg body weight.

### Mouse models of ALI

ALI induction was performed in 6–8-week-old age-matched C57BL/6 J male mice (*n* = 10–12 per group) by intraperitoneal injection of 3 mL/kg CCl_4_ (Sigma-Aldrich Trading Co., Shanghai, China) in coconut oil (v/v, 50%, Sigma-Aldrich). Control and negative control mice were injected with PBS and coconut oil, respectively. At 6 h after injection of CCl_4_, mice were divided into three groups: CCl_4_ group, injected with 100 µL PBS (supplemented with 2% mouse serum) through a tail vein; CCl_4_ + MSC group, injected with 5 × 10^5^ MSCs suspended in 100 µL PBS (supplemented with 2% mouse serum) through a tail vein; CCl_4_ + Fer-1 group, intraperitoneally injected with ferrostatin-1 (Fer-1, a ferroptosis inhibitor, 2.5 μmol/kg body weight). Erastin, intraperitoneal injection of erastin (a ferroptosis inducer, 30 mg/kg body weight) twice every other day, and then the mice were divided into two groups (*n* = 10–12 per group): Erastin group, injected with 100 µL PBS (supplemented with 2% mouse serum) through a tail vein; Erastin + MSC group, injected with 5 × 10^5^ MSCs suspended in 100 µL PBS (supplemented with 2% mouse serum) through the tail vein. The mice were sacrificed 48 h after MSCs injection, and serum and liver tissues were collected for subsequent analysis. The individuals caring for the animals and conducting the experiments were blinded to the allocation sequence, blinded to group allocation. Randomization was used to determine the allocation of animals to the experiments.

### Isolation and incubation of primary hepatocytes

The same ALI mouse models were established. Primary hepatocytes were collected according to the rapid two-step method using collagenase perfusion. We cultured the primary hepatocytes in William’s E Medium containing 10% fetal bovine serum (FBS, Gibco Biosciences, Waltham, MA, USA), 10 mg/mL streptomycin, 100 IU/mL penicillin, 1% ITS liquid medium supplement (Sigma-Aldrich, I3146), and 40 ng/mL dexamethasone. Human normal hepatocyte (L-02) cells (purchased from KCB) were cultured in RPMI-1640 (Gibco Biosciences, Waltham, MA, USA) containing 10% FBS.

### Lipid peroxidation, ROS, MDA, and cell viability measurements

Lipid peroxidation and reactive oxygen species (ROS) were detected by fluorescent probe C11-BODIPY^581/591^ (D3861; Invitrogen, Carlsbad, CA, USA) and 2′,7′-dichlorodihydrofluorescein diacetate (H_2_DCFDA) (D6883; Sigma-Aldrich) staining [15]. We used a kit (S0131S; Beyotime Biotechnology, Shanghai, China) to detect the hepatic malondialdehyde (MDA) content and the Cell Counting Kit-8 viability assay (Sigma-Aldrich) to determine cell viability.

### Real-time PCR analysis

Total RNA was isolated from liver tissue using TRIzol (Invitrogen) according to the manufacturer’s instructions. cDNA synthesis was carried out using the PrimeScript RT kit (Takara, Beijing, China). Quantitative real-time PCR was performed on the ABI QuantStudio-5 (Applied Biosystems) using SYBR Green Supermix (Thermo Fisher Scientific) and specific primers (Supplementary Table S1). Relative gene expression in real-time PCR was calculated as follows. First, we obtained the ΔCT value by subtracting the average CT value of the reference gene from the average CT value of the target gene. Then, the average ΔCT value in the control group was calculated and the corresponding ΔΔCT values were obtained by subtracting this average from the average value of each ΔCT value in each group. Next, the 2^-ΔΔCT^ value was calculated. All experiments were performed in triplicate, and melting curve analysis was performed to monitor the specificity.

### Western blot analysis

The following antibodies were used in WB analysis: anti-ubiquitin (ab134953; Abcam, Cambridge, UK), SLC7A11 (D2M7A; Cell Signaling, Danvers, MA, USA), anti-OTUB1 (ab175200; Abcam, Cambridge, UK), anti-CD44 (ab189524; Abcam), anti-SLC7A11 (ab175186; Abcam), anti-CD44 (ab119348; Abcam), rabbit monoclonal [EPR5702] to CD63 (ab134045; Abcam), rabbit monoclonal [EPR4244] to CD81 (ab109201; Abcam), GAPDH Monoclonal antibody (60004-1-Ig; Proteintech), Beta Tubulin Polyclonal antibody (10094-1-AP; Proteintech) and goat anti-rabbit IgG H&L (HRP) (ab6721; Abcam).

### Visualization of lipid peroxidation

Hepatocytes harvested from mice were resuspended in 500 μL PBS containing C11-BODIPY^581/591^ (2.5 μM) and incubated at 37 °C for 30 min in a tissue culture incubator. The cell membrane was stained with 1,1′-dioctadecyl-3,3,3′,3′-tetramethylindocarbocyanine perchlorate (Dil, D3911; Invitrogen, Carlsbad, CA, USA), a lipophilic membrane dye. The nuclei were stained with 4′, 6-diamidino-2-phenylindole (DAPI, D21490; Invitrogen, Carlsbad, CA, USA). Finally, cells were washed twice with PBS, strained through a 70 μM cell strainer, and analyzed by laser confocal microscopy.

### Immunofluorescence staining and co-immunoprecipitation

We used slides containing mouse liver tissues or primary hepatocytes. The slides were incubated with primary antibodies at 4 °C overnight. The following day, after incubation with the corresponding secondary antibody, the nuclei were stained with DAPI before observing by confocal laser microscopy. Co-immunoprecipitation analysis was carried out using the Pierce Co-Immunoprecipitation Kit (26149; Thermo Fisher) according to the manufacturer’s instructions.

### MSC-Exo labeling and tracking in mice

The details of MSC-Exo labeling and tracking procedures were provided in the supplementary materials.

### Statistical analysis

The results were presented as means ± standard deviation (SD) and were analyzed by GraphPad Prism software (GraphPad Software Inc., San Diego, CA, USA). The student’s *t*-test was performed to compare the values between the two groups. We performed one-way ANOVA followed by Tukey’s post hoc test to compare data obtained from multiple groups. *p < 0.05 and **p < 0.001 were considered to indicate statistical significance.

The procedures for isolation and culture of mouse MSCs, WB analysis, hydroxyeicosatetraenoic acids detection method by ultra-performance liquid chromatography-tandem mass spectrometry (UPLC-MS/MS) and preparation of CCl_4_-induced hepatocytes injury were described in the supplementary materials.

## Supplementary information

Supporting information

Figure. S1

Figure. S2

Figure. S3

Figure. S4

Figure. S5

Figure. S6

Supporting table S1

Reproducibility checklist

## Data Availability

All data generated or analyzed during this study are included in this article.
