# Supplementary material for: Mesenchymal stem cells protect against ferroptosis via exosome-mediated stabilization of SLC7A11 in acute liver injury
Source: Cell Death Dis. 2022 Mar 26;13(3):271. doi: 10.1038/s41419-022-04708-w (PMC8960810; doi:10.1038/s41419-022-04708-w)
Supplement: Supplementary file 1 — Supporting information [file 41419_2022_4708_MOESM1_ESM.docx]

**Supporting information**

**Methods**

**Preparation of mesenchymal stem cells**

The humerus, tibia and femur of C57BL/6 pup mice were removed, the bone marrow cavity was rinsed and cut into pieces, and bone slices were transferred to digestive solution containing type II collagenase for oscillation digestion at 37℃ for 1 h. The upper digestive fluid was absorbed and discarded, and the bone slices were cleaned repeatedly with culture solution. The bone slices were inoculated into a 10 cm plate containing complete culture solution and incubated at 37℃ with 5% CO_2_ for 3 days. On the third day, the fresh medium was replaced and cultured in the incubator at 37℃ with 5% CO_2_ for another 2 days. Finally, we can find the colonies of cells, which are primary MSCs, crawling out of the bone fragments. We have obtained a patent for this technology. The patent number is ZL 2017 1 0628074.3.

**Western blot analysis**

Total protein was extracted from fresh liver tissue using Total Protein Extraction Kit for animal Cultured Cells and Tissues (Cat No. SD-001/SN-002; Invent Biotechnologies). Protein concentration was measured using BCA protein assay kit (P0012S; Beyotime). Next, a total of 20-30 µg protein of each sample was first resolved by a 12% sodium dodecyl sulfate-polyacrylamide gel electrophoresis (SDS-PAGE), then transferred to a nitrocellulose membrane. Subsequently, the membranes were blocked with 5% BSA and incubated with the primary antibody at 4℃ overnight. After incubation with secondary antibodies, the signals on the membranes were visualized by SuperSignal™ West Pico PLUS Chemiluminescent Substrate (34577; ThermoFisher). The following antibodies were used: anti-Ubiquitin (ab134953; Abcam), xCT/SLC7A11 (D2M7A; Cell signaling), anti-OTUB1(ab175200; Abcam), anti-CD44 (ab189524; Abcam), anti-xCT (ab175186; Abcam), anti-CD44 (ab119348; Abcam), Histone H3 (D1H2) XP® Rabbit mAb (44999; Cell signaling), Rabbit monoclonal [EPR5702] to CD63 (ab134045; Abcam), Rabbit monoclonal [EPR4244] to CD81 (ab109201; Abcam), Goat Anti-Rabbit IgG H&L (HRP) (ab6721; Abcam).

**Measurement of ROS, MDA and cell viability**

We selected a kit (S0131S; Beyotime) to detect the content of hepatic malondialdehyde (MDA) and a GSH assay kit (703002; Cayman Chemical, Arbor, USA) to measure the content of glutathione (GSH). Cell Counting Kit-8 viability assay (Sigma-Aldrich) was used to determine cell viability. Reactive oxygen species (ROS) was analyzed using confocal laser microscopy with 2',7'-dichlorodihydrofluorescein diacetate (H_2_DCFDA, Sigma-Aldrich) staining.

**Condition of UPLC-MS/MS method to detect hydroxyeicosatetraenoic acids**

The chromatographic separation was achieved using an Amide C18 (2.1 × 100 mm, 1.7 µm) column, connected to ACQUITY UPLC and Xevo TQ-S Micro triple quadrupole mass spectrometer and maintained at 30℃.Samples (1µL) were injected onto the column and eluted at a flow rate of 0.4 mL/min (0-3.0 min) under gradient conditions consisting of 0.1% ammonia water (solvent A) and acetonitrile (solvent B). Gradient conditions were as follows: 0.0–0.3 min, 70% B; 0.3–1.0 min, 70-30% B; 1.0–2.0 min, 30% B; 2.0–2.3 min, 30%-70% B; 2.3-3.0 min, 70% B.  The mass spectrometer source utilized the multiple monitoring (MRM) mode with an ESI (source temperature 150℃, flow 150 L/h; de-solvation temperature 600 ℃, flow 1000 L/h) in positive ion mode at 2.5 kV. Ultra performance liquid chromatography-tandem mass spectrometry (UPLC-MS/MS) data were analyzed using Masslynx 4.1 software (Waters Corp, Milford, MA, USA).

**MSC-Exo labeling and tracking in mice**

Mouse models of acute liver injury were established as previously described. The isolated exosomes were labelled in 5 µM DiR (D12731; ThermoFisher). Labelled MSC-Exo was injected via tail vein into 8 weeks old male mice with or without acute liver injury. Untreated (injected with equal PBS) mice were selected as control. About 1-3 h post injection, all mice were sedated by isoflurane and imaged on an IVIS Spectrum (PerkinElmer). Next, mice were euthanized by cervical dislocation and organs were dissected and imaged. Excitation and emission filters at 750 nm and 800 nm were selected to detect the fluorescence.

**CCl_4_-induced hepatocytes injury in *vitro***

Before experiment, all kinds of plates were coated with collagen coating solution (125-50; Sigma-Aldrich) and dried naturally. Primary hepatocytes were seeded in 96-well plate (3×10^3^ cells/well) or 6-well plate (1×10^5^ cells/well), cells were treated with medium containing 0, 2, 4, 6, 8, 9, 10, 11, 12, and 16 mM CCl_4_ for 24 h, 48 h, 72h. Medium containing dimethyl sulfoxide (DMSO) was control. Cell viability was measured by Cell Counting Kit-8 viability assay. Next, various concentrations including 20 µg, 40 µg, 80 µg and 160 µg MSC-Exo were applied to CCl_4_-induced injury hepatocytes. Cells were collected and RNA and protein and lipid peroxidation were detected.

**Immunofluorescence**

Mouse liver slides were dewaxed and rehydrated, then submerged into pre-heated antigen retrieval solution before blocking with 5% fetal bovine serum (FBS). Next, the liver sliders were incubated with SLC7A11 antibodies at 4°C overnight. The next day after incubation with corresponding secondary antibody and nuclear staining with 4', 6-diamidino-2-phenylindole (DAPI), the slides were observed by confocal laser microscopy.

**Immunohistochemistry (IHC)**

The formalin-fixed and paraffin-embedded blocks were cut into 4 µm sections for immunostaining. The sections were incubated with anti-4 hydroxynonenal (4-HNE) (ab46545; Abcam) overnight at 4 °C. Histostain-Plus 3rd Gen IHC Detection Kit (Invitrogen Co., San Diego, CA) was applied to visualize the positive signals.

**Figure legends**

**Fig. S1 The original western blots of Figure 3.** (A) The original western blots of Figure 3B. SLC7A11 protein level was significantly down-regulated when the CCl_4_ concentration was greater than or equal to 10 mM. (B) The original western blots of Figure 3C. The SLC7A11 protein levels were decreased at 24 h, 48 h and 72 h after CCl_4_ treatment (10 mM). (C and D) The original western blots of Figure 3E. SLC7A11 protein level was reduced in CCl_4_ group compared with PBS group, but was restored by MSCs co-culture.

**Fig. S2 CCl_4_-induced decrease in SLC7A11 protein level was restored with MSC-Exo treatment.** (A) Total protein was extracted from MSC and MSC-Exo. And WB analysis was performed to detect the protein levels of CD44, CD63 and CD81. (B) Total protein was extracted from liver tissue in Control (oil), CCl_4_ and CCl_4_+MSC-Exo groups. And WB analysis was performed to detect the protein levels of CD44 and SLC7A11 in three samples of each group.

**Fig. S3 The original western blots of Figure S2.** (A) The original western blots of Figure S2A. (B) The original western blots of Figure S2B.

**Fig. S4 The original western blots of Figure 5E.** The secretion of exosomes was significantly inhibited by GW4869 in MSCs.

**Fig. S5 MSC treatment reduced the elevation of 4-HNE in CCl_4_ induced ALI.** In situ analysis of 4-hydroxynonenal (4-HNE, another marker of lipid peroxidation) in liver was performed by immunohistochemistry. (A) The induced elevation of 4-HNE by CCl_4_ was alleviated by MSC or Fer-1 treatment. However, when inhibited the release of MSC-Exo with GW4869, the reduced effect of MSCs on 4-HNE expression was weakened. (B) The induced elevation of 4-HNE by erastin was alleviated by MSC or Fer-1 treatment. But, when inhibited the release of MSC-Exo with GW4869, the reduced effect of MSCs on 4-HNE expression was weakened. (C) Statistical analysis of repeated samples in each group of figure A (n=3). (D) Statistical analysis of repeated samples in each group of figure B (n=3). Significance was calculated by one-way ANOVA with Tukey’s post-hoc test. **p < 0.05* or ***p < 0.001* indicated a significant difference between groups.

**Fig. S6 The original western blots of Figure 6.** (A) The original western blots of Figure 6A. MSC-Exo-induced recovery of SLC7A11 protein was accompanied by increasing of CD44 and OTUB1. (B) The original western blots of Figure 6B. CCl_4_ administration increased the ubiquitination of SLC7A11, while MSC-Exo treatment down-regulated the ubiquitination of SLC7A11. (C) The original western blots of Figure 6D. The endogenous CD44 and OTUB1 proteins were co-precipitated by a SLC7A11-specific antibody. (D and E) The original western blots of Figure 6E and F. Endogenous SLC7A11 was co-precipitated by a CD44-specific and OTUB1-specific antibody, respectively.
