# Supplementary figures and images for: Mesenchymal stem cells protect against ferroptosis via exosome-mediated stabilization of SLC7A11 in acute liver injury
Source: Cell Death Dis. 2022 Mar 26;13(3):271. doi: 10.1038/s41419-022-04708-w (PMC8960810; doi:10.1038/s41419-022-04708-w)

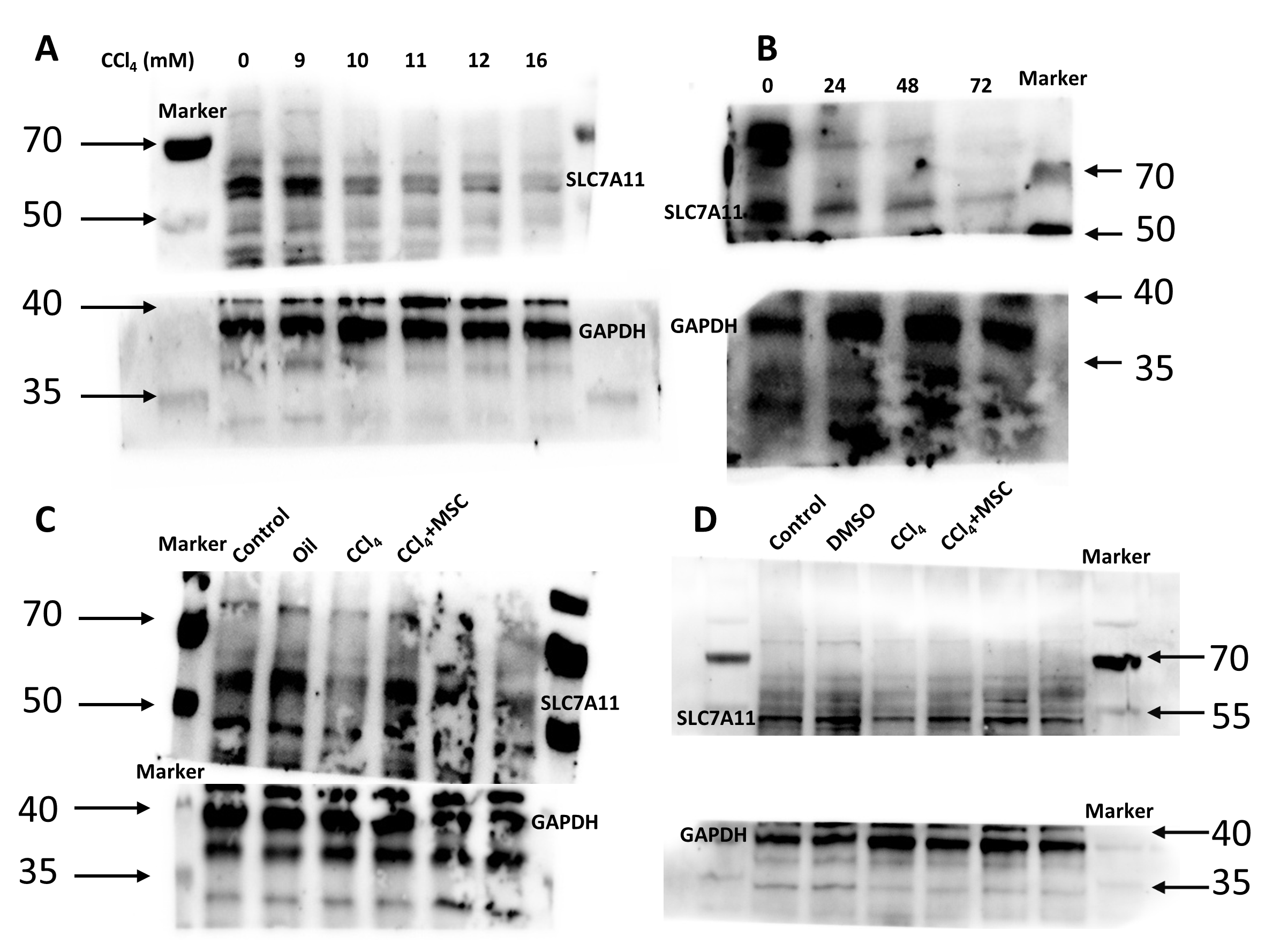

Supplement: Supplementary file 2 — Figure. S1 [file 41419_2022_4708_MOESM2_ESM.tif]

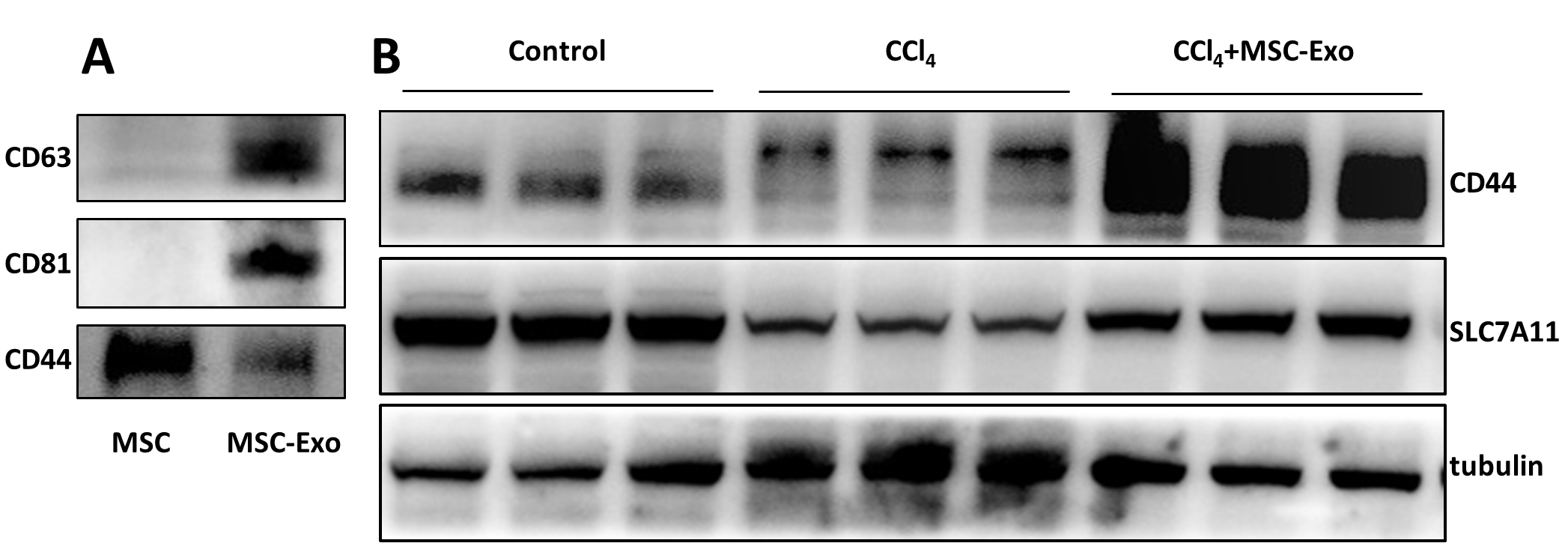

Supplement: Supplementary file 3 — Figure. S2 [file 41419_2022_4708_MOESM3_ESM.tif]

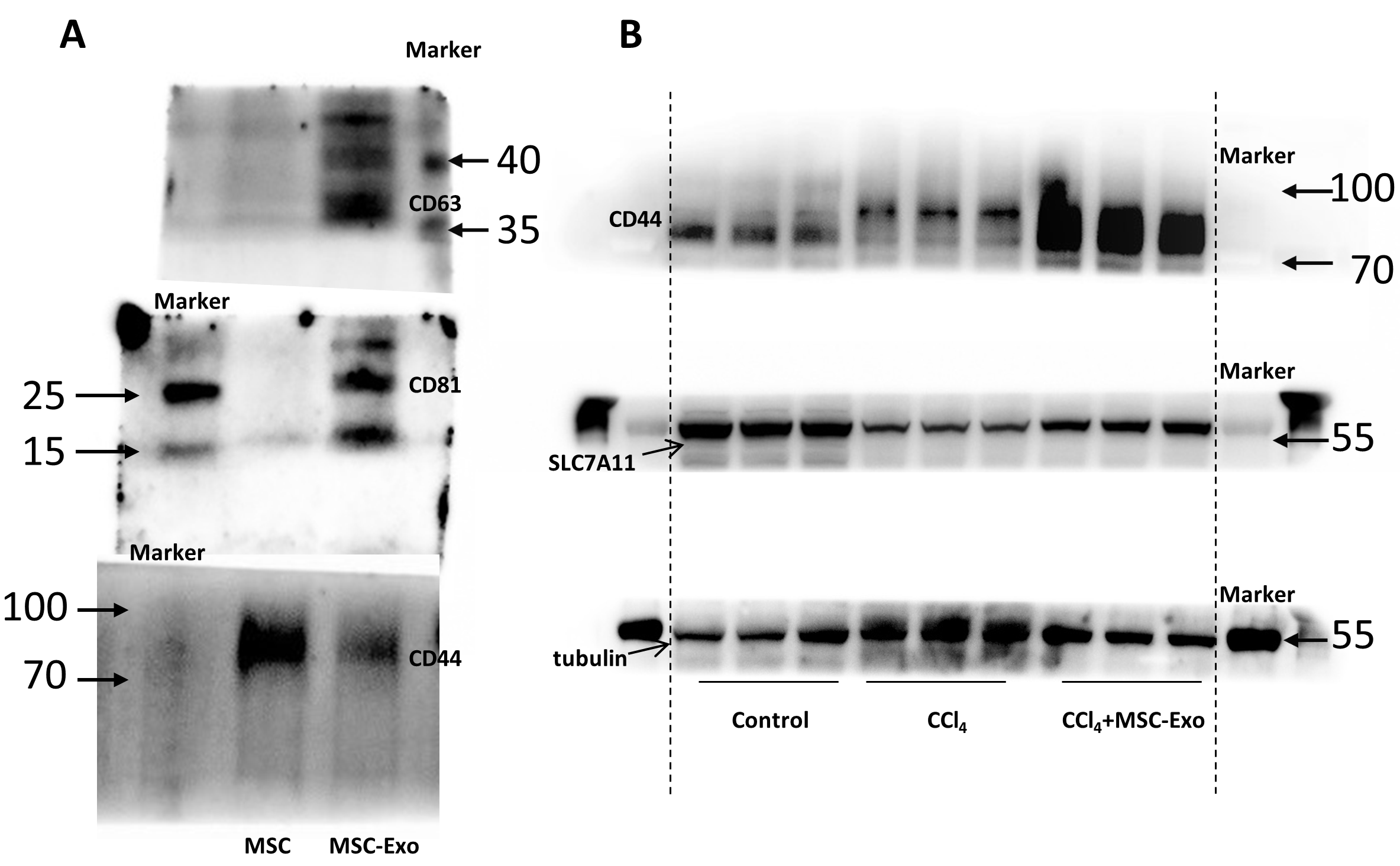

Supplement: Supplementary file 4 — Figure. S3 [file 41419_2022_4708_MOESM4_ESM.tif]

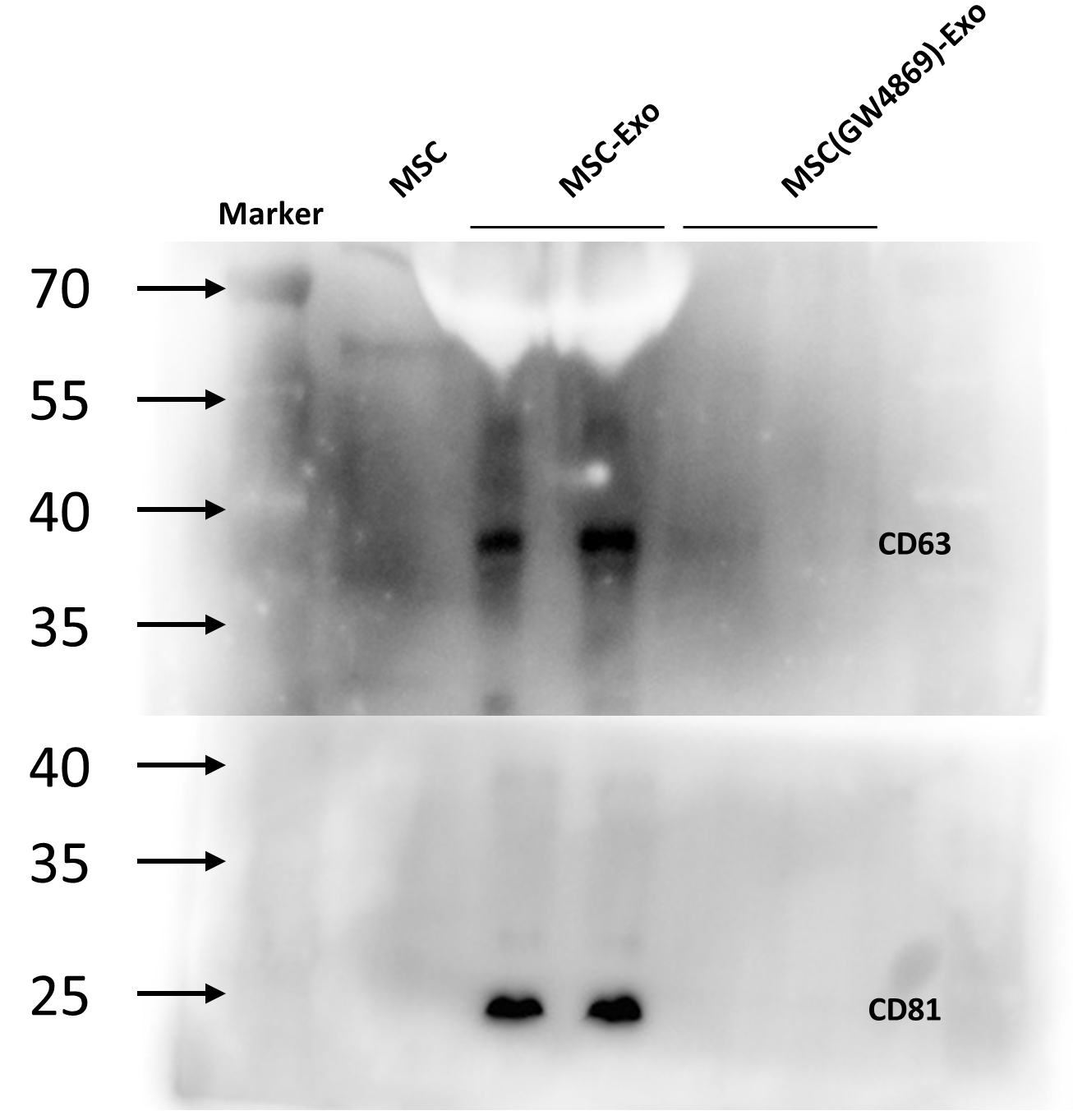

Supplement: Supplementary file 5 — Figure. S4 [file 41419_2022_4708_MOESM5_ESM.tif]

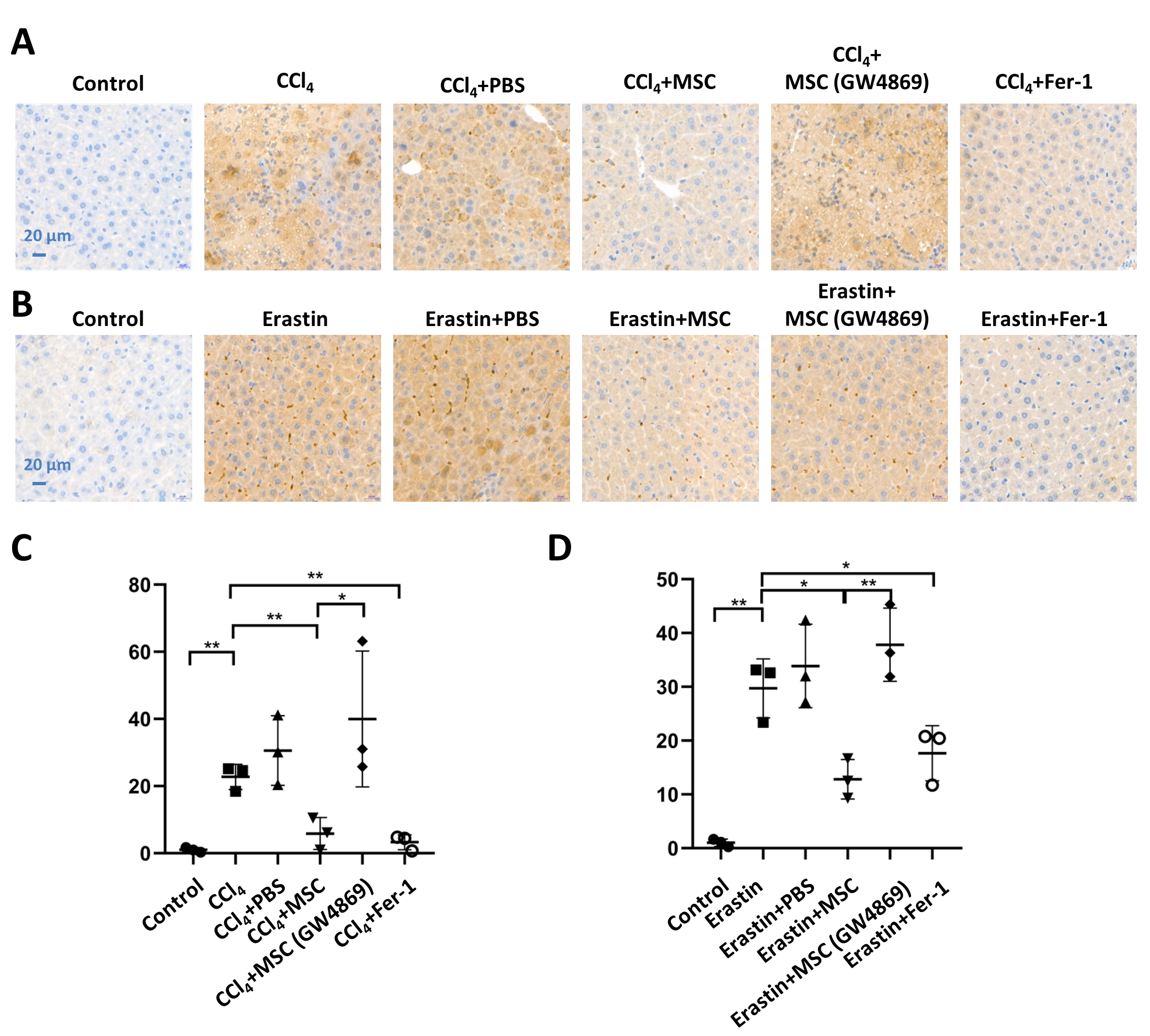

Supplement: Supplementary file 6 — Figure. S5 [file 41419_2022_4708_MOESM6_ESM.tif]

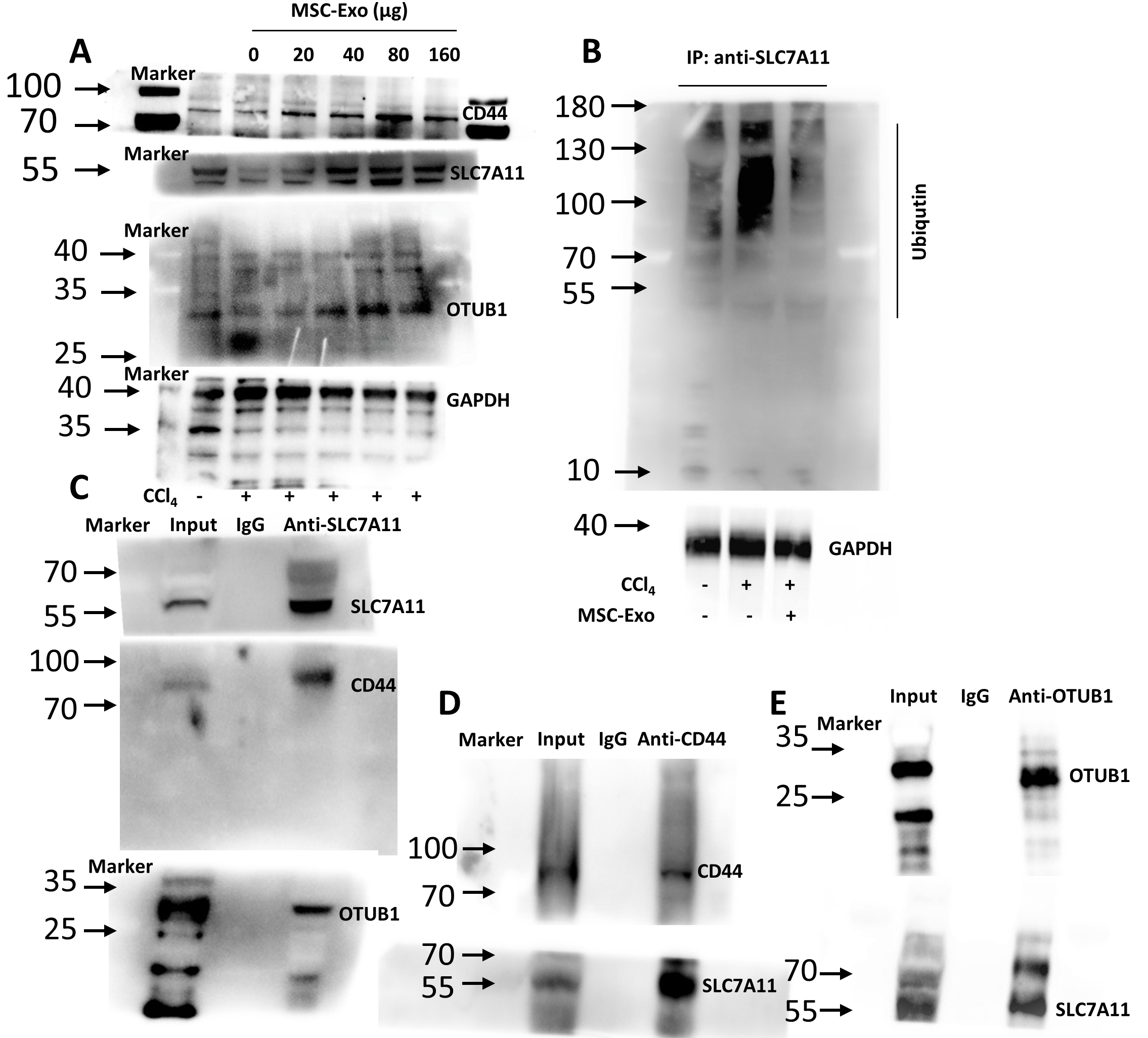

Supplement: Supplementary file 7 — Figure. S6 [file 41419_2022_4708_MOESM7_ESM.tif]
