# Supplementary material for: Mesenchymal stem cells protect against ferroptosis via exosome-mediated stabilization of SLC7A11 in acute liver injury
Source: Cell Death Dis. 2022 Mar 26;13(3):271. doi: 10.1038/s41419-022-04708-w (PMC8960810; doi:10.1038/s41419-022-04708-w)
Supplement: Supplementary file 8 — Supporting table S1 [file 41419_2022_4708_MOESM8_ESM.docx]

**Table S1 List of primer sequences**

| Gene Name | Primer sequences |
| --- | --- |
| *Ptgs2*-F | TCCTGGAACATGGACTC |
| *Ptgs2*-R | GCTCGGCTTCCAGTATTGAG |
| *15-LOX*-F | GCGACGCTGCCCAATCCTAATC |
| *15-LOX*-R | CATATGGCCACGCTGTTTTCTACC |
| *12-LOX*-F | AGTGACACCGATGTGAAGGAG |
| *12-LOX*-R | CTCTCAGATGGTCACACTG |
| *5-LOX*-F | ACTACATCTACCTCAGCCTCATT |
| *5-LOX*-R | GGTGACATCGTAGGAGTCCAC |
| *GAPDH*-F | AGGAGAGTGTTTCCTCGTCC |
| *GAPDH*-R | TGCCGTGAGTGGAGTCATAC |
